# Assessment of Sensory and Texture Profiles of Grape Seeds at Real Maturity Stages Using Image Analysis

**DOI:** 10.3390/foods10051098

**Published:** 2021-05-15

**Authors:** María Jesús Cejudo-Bastante, Francisco J. Rodríguez-Pulido, Francisco J. Heredia, M. Lourdes González-Miret

**Affiliations:** Food Colour and Quality Laboratory, Facultad de Farmacia, Universidad de Sevilla, 41012 Sevilla, Spain; mjcejudo@us.es (M.J.C.-B.); heredia@us.es (F.J.H.); miret@us.es (M.L.G.-M.)

**Keywords:** grape seeds, ripening, DigiEye^®^, sensory analysis, texture analysis

## Abstract

The usefulness of digital image analysis in estimating sensory attributes of grape seeds in relation to maturation level was evaluated for the first time. Seeds from Syrah grapes harvested throughout the ripening period were grouped according to maturity using the DigiEye^®^ system. The discriminant ability, homogeneity, repeatability, and uniformity of a sensory panel were assessed after training on grape seeds. The aim was to evaluate the use of digital image techniques in order to accurately establish the maturity level of grape seeds, based on sensory and textural features. All sensory attributes (color, hardness, cracking, vegetal, bitterness and astringency) showed significant (*p* < 0.05) correlations with the chemical maturity stage. Color and vegetal (sensory attributes), together with deformation energy (instrumental texture parameter) (De), allowed for the classification of the seeds into four real maturity stages, hence their usefulness as grape seed ripening indicators. Significant (*p* < 0.05) and high-correlation factors were also found between instrumental and sensory attributes. Therefore, digital analysis can be a useful tool to better define the maturity stage in the vineyard, and to dispose of grape seeds with well-defined sensory profiles for specific oenological applications. This could help to determine the optimal harvest date to manage winemaking, in order to produce high quality wines in warm climates.

## 1. Introduction

In warm regions, the stressful climate conditions make it difficult to produce high-quality red wines characterized by high intensity and stable color. This is due to a lag between the technological and the phenolic maturation processes of grapes, compared to how they traditionally occurred in colder viticulture zones [1]. Hence, different levels of both phenolic and sugar maturity exist at the moment of harvesting [2]. The seeds remain unripe, and thus provide low quantities of copigments, hampering the copigmentation phenomena, which contribute to color stabilization [3]. As a result, a color fade after some months of storage occurs in either bottle or barrels, which leads to a negative impact on the sensory acceptance of the wines. Therefore, the maturity of the seeds at harvesting is of great importance when aiming to produce high-quality red wines, especially in the areas most affected by the effects of climate change.

Adding oenological tannins is a normal procedure carried out by winemakers to improve wine quality in cases of insufficient extraction of phenolic compounds from grapes [4], which is a common situation with wines elaborated in warm climates. In fact, some studies have found that wines obtained from long maturation grapes (delaying the harvest date) showed increases in their sensory quality, which leads to an increase in the total flavonoid content of the seeds [5]. In this sense, by achieving the objective of this work (to accurately establish the sensory profiles of grape seeds at each maturity stage), winemakers could select grape seeds at the desirable maturity stage to be added to wine, depending on the sensory attributes that they want to enhance. Consequently, the wine industry would contribute the additional benefit of reducing the environmental impact produced by grape pomace, by reusing the grape seed by-product [6].

Grape seeds show great heterogeneity, even at the same stage of grape maturation, which implies a wide range of chemical and sensory profiles [7]. This variability makes it difficult to establish a defined sensory profile of seeds during the ripening process, based on which, the optimum harvest date could be determined. If an erroneous date is chosen, the final quality of red wines can be negatively affected. This is the main problem with grapes cultivated in warm regions. To overcome these disadvantages, it could be very interesting to apply advanced techniques that permit obtaining immediate information concerning a wide number of grape seeds at the same time in order to reduce the problem of representability. In this respect, our research group has developed new methodologies, based on imaging techniques, for evaluating the chromatic heterogeneity, which can be applied to establish homogeneous maturity stages of grape seeds cultivated in a warm climate [8,9,10]. Food products usually have specific sizes and shapes, and it is difficult to measure optical properties such as color, appearance, and other characteristics. In this sense, the DigiEye^®^ imaging system is not a traditional colorimetric instrument, because it can evaluate not only color but also other features related to appearance, by acquiring the optical properties from each pixel [10].

Establishing sensory profiles of grape seeds cultivated in a warm climate at each ripening stage is relevant to deciding upon the appropriate time to harvest. The application of digital imaging with that purpose is quite challenging. Some reports about the relationships between image analysis and sensory evaluation have been previously carried out in some foodstuffs, such as long-ripening hard cheeses [11], packaged bratwursts [12], pesto sauces [13] or the Italian taralli product [14]. However, to the best of our knowledge, there are no scientific studies that point out the relationship betwen image analysis and organoleptic attributes (sensory and texture) for establishing real grape seed maturity. Thus, this research work aims to evaluate the possibility of using digital imaging techniques to establish an accurate characterization of grape seeds based on sensory and textural features. The study was carried out on grapes grown in the Condado de Huelva D.O., a warm climate area in southwestern of Spain.

Results from these oenological applications of image analyses could have twofold benefits. The first one is the possibility, for the first time, of accurately establishing a global characterization of the sensory profiles of grape seeds at each actual ripeness level. Thus, wineries could have grape seeds sensorially characterized in order to improve certain characteristics of red wines; at the same time, this practice enables the circular production by reusing the oenological by-product and avoids the legal restrictions regarding the use of other non-grape-derived products for similar purposes. In addition, the second benefit is establishing the sensory attributes and texture parameters that best define the ripening stage, which can be used to better fix the ripening stage in the field. Thus, the optimum harvest date could be set without having to first taste the grape seeds, circumventing the inherent subjectivity and unpleasantness of the sensory evaluation of grape seeds. To carry out the study, a complete training of the sensory panel was accurately performed.

## 2. Materials and Methods

### 2.1. Sample Collection

The sample collection was composed of forty batches of Syrah grapes collected in Condado de Huelva Denomination of Origin (Spain), a typically warm-climate region according to the Köppen climatic classification [15]. Five ripening dates were considered during the berry maturation (from veraison until over-ripeness); 25, 15, and 7 days before the harvesting date, the day of harvesting, and 15 days after the harvesting date. The sampling was carried out in the central zone of the vineyard, picking up one bunch per stock and sampling two different stocks from four marked rows (a total of eight bunches per sampling date). This procedure allowed for the limiting of the grape seeds heterogeneity. All grape seeds (approximately 1500 seeds) were removed from the pulp, left to dry at room temperature for 2 h, and kept in a freezer. The grape seeds were then classified according to maturation stages using the DigiEye^®^ imaging system.

### 2.2. Image Acquisition and Appearance Measurements

The DigiEye^®^ imaging system, based upon the calibrated digital camera, was used [16]. This device consists of a closed illumination box, specially designed by VeriVide Ltd. (Leicester, UK), and a digital camera (10.2-megapixel Nikon^®^ D80 with a Nikkor^®^ 35 mm f/2D objective) (Aquateknica AQ instruments, Valencia, Spain). It was calibrated with the DigiTizer (VeriVide Ltd., Leicester, UK) color chart with the objective of characterizing the camera response by relating its RGB signals to CIE specifications. The cabinet is equipped with two CIE D65 standard illuminant emulators, which illuminate the samples consistently under stable lighting conditions [17]. Lamps were switched on at least ten minutes before the measurements. Different sets of 96 individual grape seeds were recorded at once. This amount was the result of the 12 × 8 grid that allowed the identification of each seed within the image. For obtaining morphological and appearance parameters, and the CIELAB coordinates from RGB color space data, the software DigiFood^®^ [18] was used. The application of the methodology and the algorithm for fitting point clouds into ellipsoids were developed on MATLAB R2019a [19]. After the DigiEye^®^ analysis, all samples were frozen at −20 °C for further analyses.

### 2.3. Sensory Analysis of Grape Seeds

Twenty members of the laboratory staff (13 women and 7 men, from 30 to 55 years old), with previous experience in sensory evaluation of grape-derived products, participated in the study. Judges were recruited according to their motivation and availability during a nine-month study in two sessions per month.

After some sessions elaborating the profile sheet based on training sessions to develop a common vocabulary, wine professionals generated the final list of attributes evaluated by touch, texture in mouth, visual properties and taste, including color, hardness, cracking, vegetal, bitterness and astringency. As the color could influence the classification, expert tasters were advised to assess all parameters independently of the color.

All sensory attributes were selected, defined, and established by consensus of the panel members, as well as the number of seeds to put into the mouth (three) and the number of chews (four) to perform for a full sensory evaluation.

The sensory panel was first accurately evaluated and, after training sessions, the panel, which was composed of judges that displayed appropriate discriminant ability and repeatability, exhibited homogeneity and uniformity. The complete sensory analysis procedure is described in Results and Discussion section.

### 2.4. Instrumental Texture Analysis

The compression test was performed using a TA.XT Plus Texture Analyzer (Stable Micro System, Godalming, UK) equipped with a HDP/90 platform. Weight calibration of equipment was carried out with a 5 kg load cell, and previous calibrations of force and distance were performed with a 30 kg load cell. The compression test was performed for each entire grape seed, using a 20 mm cylindrical aluminum probe. Grape seeds were lyophilized for the analysis. The test parameters were as follows: speed pre-test, 1.0 mm/s; speed test, 1.0 mm/s; speed post-test, 10 mm/s; distance, 10.0 mm; activation force, 5.0 g; trigger type and auto force, and 500 points per second. The compression test ended when the seed reached 50% of its height. The probe begins to move at a constant speed. When it touches the seed, the graph starts to be registered. During the first stage, a linear behavior is observed until the seed no longer withstands the stress and breaks (crunches), rapidly decreasing the force measured by the cell. Although the seed is firstly broken, the load cell continues to compress it, increasing the load again and appearing successive peaks resulting from subfragmentation. When the cell reaches 50% of the initial thickness, it is retracted and the test ends. As both the probe and the sample surface are flat, the position of the seed is not relevant.

**Figure 1:**
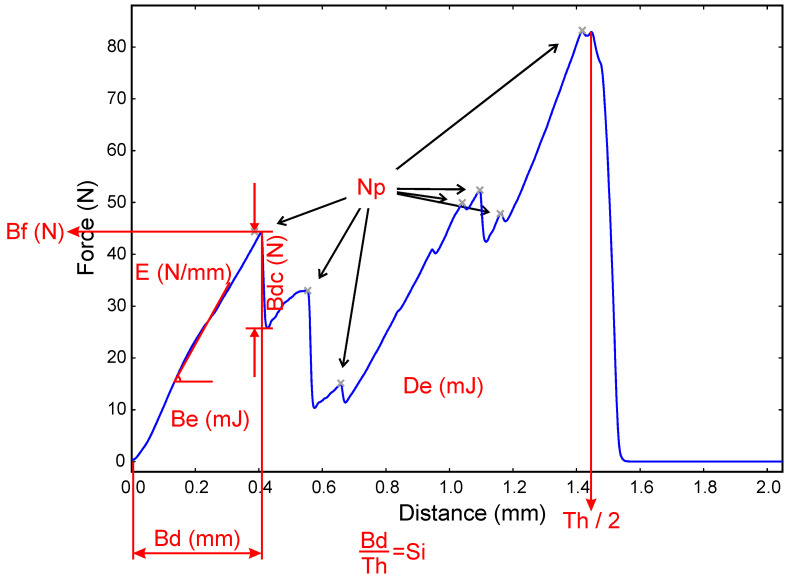
Force–distance curves and the extracted parameters of grape seeds. Np, number of peaks; Bf, breaking force; Bdc, breaking decline; E, elasticity; Be, breaking energy; De, deformation energy; Th, thickness; Bd, breaking distance; Si, strain index.

Texture Exponent software was used for data acquisition. The raw data were recorded in graphs, in which the abscissa corresponds to the distance (mm) covered by the probe from the test starts, and the ordinate is the force (N) detected by the load cell. Force–distance curves were analyzed (Figure 1) and different parameters were extracted and studied (Table 1). MATLAB R2019a [19] was used for performing texture analyses.

### 2.5. Statistical Analysis

All statistical analyses were performed using Statistica v.8.0 software [20]. Analyses of variance (ANOVA) were applied to establish whether mean values of the sample data differed significantly (*p* < 0.05) from each other, and to evaluate the panel performance. The mean values of each set of samples were compared by the Student–Newman–Keuls test (SNK) at a significance level of *p* < 0.05. Principal component analysis (PCA) was carried out on the normalized data set to evaluate the panel performance, and to obtain summarized and synthesized information in order to better understand the maturity stage effects. Correlation matrix was carried out for scrutinizing eventual relationships among parameters.

## 3. Results and Discussion

### 3.1. General Parameters

Mean values of weight (g), calibre (cm), veraison (%), sugar content (°Baumé), pH and acidity (mg/L expressed as tartaric acid) of grapes for all sampling points are shown in Table 2.

### 3.2. DigiEye^®^ Classification

**Figure 2:**
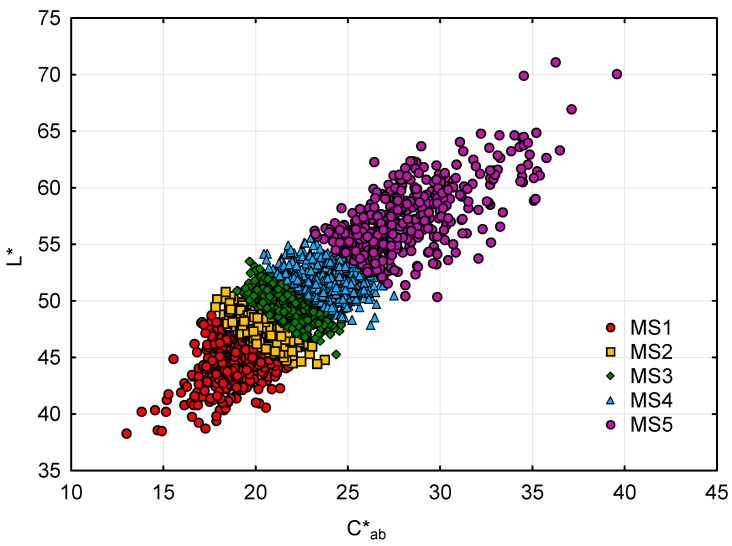
Scatterplot of L* against C*_ab_ categorized by percentiles.

The classification using the DigiEye^®^ system was developed using percentiles, which guaranteed the same number of grape seeds for each homogeneous group. The DigiEye^®^ imaging system has been previously validated as the procedure for establishing the real maturity stages of grape seeds, taking into consideration CIELAB coordinates from RGB color space [9,10]. Lightness (L*) and chroma (C*_ab_) were chosen and measured as classification criteria (Figure 2) because they are the parameters which change the most during the maturation period [9] (Figure S1).

As a result, grape seeds were allocated into five predefined groups (from MS1 to MS5), corresponding to different maturation stages. Once every grape seed was assigned to a ripening stage, all seeds were distributed into two different sets, each containing all maturation stages: one set composed of approximately 500 grape seeds, which was used for training and sensory evaluation, and another set composed of 50 grape seeds, which was used for the instrumental analyses of texture.

### 3.3. Sensory Descriptive Analysis Procedure

#### 3.3.1. Training Sessions

Ten one-hour training sessions were carried out from October to April, which were organized as follows. In the first three training sessions, recognition tests were carried out. Panellists were asked to recognize the odors and tastes of standards related to the parameters that would be evaluated in the sensory sessions (tannic acid, *cis*-hexenal:IBMP and caffeine) (Sigma-Aldrich, St. Louis, MO, USA). The attributes, definitions, standards, and their concentrations are expressed in Table 3. The standards used and their concentrations are comparable to those reported by Le Moigne et al. [21] and Ramos-Pineda et al. [22]. For each sample, panellists took 5 mL, smelt it or tasted it and spat it out (depending on the standard sample), without knowing the nature of the samples. The subsequent sessions were carried out to perform the ranking tests of each odor and taste standard. For that purpose, different dilutions from the standard concentration used in the third session of the previous standard recognition were used (Table 3). Then, panellists were asked to rank a group of samples in ascending order (ISO 8587:2010). Once the training sessions finished, those candidates that were able to recognize the samples and to rate the intensity of their corresponding attributes were selected for the panel (seventeen panellists).

#### 3.3.2. Evaluation of Panellist Performances

The panel was evaluated to test and prove its reliability. Coded grape seeds were randomized and then presented to the panellists in each session, to limit carry-over effects and memory biases. The samples evaluated in each session were the same for all judges, as the order was randomized across sessions. This design also allowed for the reproducibility and performance consistency of the judges to be determined.

For the sensory analysis, the grape seeds from each maturity stage were previously classified using the DigiEye^®^ system. Each sample consisted of three grape seeds, which was considered an adequate number for a good perception of the sensory attributes. The seeds were presented on a white plastic plate 8 h after they were taken out of the freezer. Tasters were provided with mineral water and plain crackers to cleanse the palate between samples, waiting 3 min before tasting the next one. A structured ten-point rating scale (ISO 4121:2006), anchored at the end, was used to quantify all the attributes. Judges were asked to first evaluate the color (from green = 0 to brown-black = 10). Hereafter, panellists were asked to put the seeds into their mouths (3 seeds at once), chew them four times, and then assess the rest of the attributes (from little = 0 to 10 = much). At the end of assessing all samples, panellists were asked to rank the coded samples according to the maturity level, and to specify the attributes that lead to this sorting (ISO 8587:2010). The samples evaluation was conducted in 20 min sessions. At the end of every session, the panel leader led a 30 min meeting with all the panellists to unify criteria.

Eight sensory evaluation sessions were developed. Three-way analysis of variance (ANOVA) was applied to each attribute to assess the panel’s performance, considering the sample, session, and judge effects, as well as the interactions among them. All attributes were considered as variables, and the scores of each maturity stage and judge were considered as cases.

**Figure 3:**
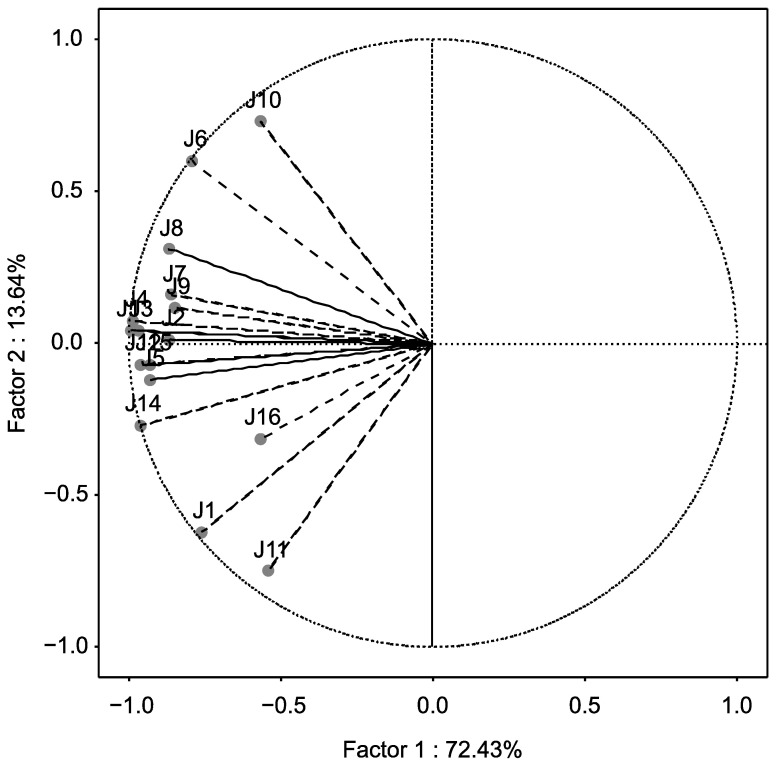
Projection of the variables on the factor-plane extracted by PCA corresponding to the vegetal attribute.

For assessing the homogeneity of the panel, the interaction judge × sample was taken into account, which was significant (*p* < 0.05) for all attributes, except for the color. PCA was performed for each attribute, considering the judges as variables and the samples as cases, to test whether the interaction was due to disagreement between judges or to scale effects [23]. The representation of the two principal components evidenced that the interaction was due to scale effects, since all judges were situated on the same dimension in the plane with a high variance (72.43%) (Factor 1) (Figure 3).

Therefore, all attributes were considered. The more disperse position of some panellists (judges 1 and 11 were opposed to judges 6 and 10) on the second dimension in the plane (Factor 2, related to the sample × session interaction) could be explained by using different descriptors to discriminate samples, and by the heterogeneity of the samples, more than by the training. It must be considered that some punctual visual differences among the grape seeds were naturally observed, which is why authors considered three grape seeds as one tasting sample. However, the discriminant ability of the panel was demonstrated based on the significance (*p* < 0.05) for all attributes of the sample effect (Table 4) [21]. Furthermore, the scores of the session and judge × session interaction demonstrated the panel uniformity and repeatability along with sessions.

According to the results achieved, those assessors with the utmost scores of variance and judgment dispersion were discarded from the panel. 

#### 3.3.3. Accurate Evaluation of the Sensory Parameters at Each Maturation Stage

In the light of the fact that all attributes showed significant (*p* < 0.05) differences (sample effect) (Table 4), a more detailed analysis of variance was performed to determine the best sensory characteristics to discriminate between ripening levels. For that purpose, mean scores of each attribute at each maturity stage were compared using SNK.

**Figure 4:**
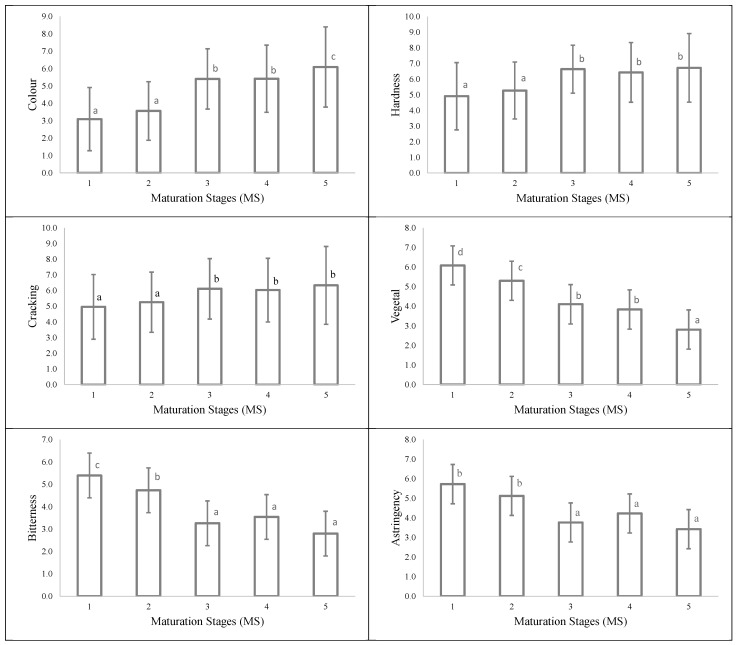
Evolution of seed sensory attributes on a 0–10 scale of all maturity stages (MS). Means and standard deviation. Different letters indicate significant (*p* < 0.05) differences according to Student–Newman–Keuls.

Figure 4 shows the evolution of the means and standard deviation of each sensory attribute of the grape seeds according to the maturity stages, previously established by using the DigiEye^®^ system.

As can be seen, the change in color at veraison from green (from 0 to 3) to brownish (around 5) was reflected in the increasing values throughout the ripening. In parallel, the scores of hardness and cracking also became higher, probably related to the dehydration of the outer integument of the seed and a lignification of the medial tissue occurring during ripening [24,25]. Moreover, the vegetal, bitterness, and astringency values decreased from stage 1 of maturity onwards, similarly to the data reported by Bautista-Ortín et al. [26]. From stage 5 onwards, grape seeds were perceived to have lower scores in astringency, vegetal, and bitterness, probably supported by the decrease in the amount of extractable catechins and tannins during ripening, due to combination with other substances, which are more difficult to extract.

On the other hand, the vegetal attribute was able to significantly (*p* < 0.05) differentiate among four maturity stages, and color and bitterness among three stages (Figure 4). Correlation matrixes were performed to scrutinize the relationship between sensory attributes. Positive and significant (*p* < 0.05) correlations were found between vegetal and bitterness (R^2^ > 0.70), and vegetal and astringency (R^2^ > 0.60) (data not shown). These results could indicate that the vegetal and color attributes play a major role as indicators in the maturity differentiation, and therefore could be the most responsive sensory attributes to follow up the grape seed ripening. In fact, color and vegetal parameters fitted to a linear regression with the maturity stage (R^2^ > 0.90) and allowed a high accuracy classification of the seeds.

### 3.4. Evolution of Instrumental Textural Parameters along the Grape Seed Ripening

The evaluation of the mechanical and physical characteristics of grape seeds was performed using texturometry. Prior to scrutinizing the data, the relationships between parameters were evaluated to eliminate those variables strongly correlated with others that were more useful or easier to understand from a texturometric point of view. For that purpose, correlation matrixes were performed among parameters, and R coefficients > 0.80 were found for the pairs breaking energy (Be) and breaking force (Bf), breaking energy (Be) and breaking distance (Bd), and strain index (Si) and area ratio (Ar) (data not shown).

Table 5 shows the mean scores and standard deviation of instrumental textural parameters of the grape seeds, according to the maturity stages (MS), which were previously established using the DigiEye^®^. The effect of the ripening on the instrumental textural parameters was investigated, and SNK was applied to scrutinize the significant (*p* < 0.05) differences among them.

As can be seen, slight tendencies were observed depending on the maturity stage, taking into account that many instrumental texture parameters became steady after veraison, as previously affirmed Río Segade et al. [27]. Thus, thickness (Th), strain index (Si), and area ratio (Ar) attributes evolved similarly, showing significant (*p* < 0.05) differences between MS1 and the rest of the maturity stages, probably because the initial grape seeds were smaller and easier to deform than the rest. Likewise, grape seeds become harder as maturity progresses, possibly due to the lignification of tissues, where the cell wall becomes thickened and impregnated with lignin [24,25]. Consequently, grape seeds required a higher force to be broken (Bf), and more energy to be deformed or broken (Be). Among all, deformation energy (De) appears to be the most influencing textural parameter for maturity stage discrimination, in agreement with Letaief et al. and Rolle et al. [28,29], as emphasized by the significant (*p* < 0.05) differences, enabling the grape seeds to be distinguished and grouped into three maturity stages. Therefore, these results evidenced that the DigiEye^®^ image system enabled the determination of the ripening stage in relation to the measurement of the energy applied to compress the seed up to 50% of its thickness (De).

### 3.5. Correlation between Sensory and Instrumental Analyses of Grape Seeds

**Figure 5:**
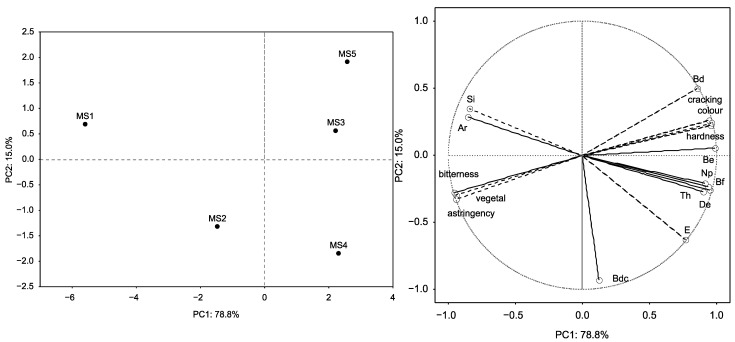
Distribution of samples in the plane defined by the two first discriminate functions by maturity stage and projection of the variables on the factor-plane extracted by PCA.

Non-supervised pattern recognition statistical analysis (principal component analysis) was applied to the data set (instrumental and sensory parameters) in order to establish the parameters more related to the maturity differentiation. Four main principal components (PCs) arose according to Kaiser’s criterion (eigenvalues > 1), which explained 100% of the variance. Figure 5 shows a biplot with the projection of the samples and the parameters to the plane defined by the two principal components (PC1 and PC2), which explained 93.8% of the variance (virtually all the variations of instrumental texture parameters and sensory attributes).

As can be seen, a separation by ripening was achieved. On one hand, color, cracking and hardness (both instrumental and sensory for the latter), together with other mechanical parameters, were found at the positive end of the first axis and were therefore positively correlated with the MS3, MS4 and MS5. On the other hand, the instrumental Si and Ar, and the sensory vegetal, bitterness and astringency parameters were found at the negative end of the first axis, positively correlated with MS1 and MS2.

To determine the relationship between instrumental and sensory information or data, a correlation matrix was performed. Except for Bdc, strong correlations were achieved (r values from 0.60 to 0.99), mainly between Np, De, Th and the related parameters Bf, Be and Bd, with all sensory attributes (Table 6). This result indicates that it is possible to describe sensory variations through instrumental measurements, and it highlights the effectiveness of texture analysis in identifying substantial differences between the sensory profiles of grape seeds throughout the ripening process. However, at the same time, it appears to be less effective in detecting small changes linked to advanced maturity stages of grape seeds. The labelling of seeds was performed regarding their appearance instead of the date of sampling. It was made this way because of two reasons. As it is stated in the Introduction, there is a great heterogeneity in the seeds in the same stage of maturation. Moreover, previous studies have proved the relationship between appearance and composition. Although this labelling was made judiciously, there are more phenomena involved. Chemical changes occur quickly in the first stages of maturation. This means that the differences in composition will be greater between the first stages than between the last ones. Therefore, it makes sense that it is more difficult to detect small changes in these advanced maturity stages. The good agreement between image analysis and sensory analysis and instrumental parameters suggests that these methodologies could be applied in combination for monitoring grape seed maturity.

## 4. Conclusions

The DigiEye^®^ system has been accurately assessed as an innovative technique to monitor the sensory attributes and textural parameters of grape seeds throughout the ripening process, based on digital image analysis. To bring this fact to fruition, instrumental textural parameters were measured and sensory analysis of seeds at different maturity levels was performed, considering maturation levels such as the phases established in this article by image analysis. The implementation of DigiEye^®^ has allowed the accurate characterization, for the first time, the sensory profile and the instrumental textural properties of grape seeds at different real maturity stages, achieving a good differentiation of grape seeds over ripeness. Color and vegetal attributes were found to be the best sensory indicators to discriminate, and the deformation energy parameter was useful in following up the textural properties during grape ripening, representing new maturity markers for the wine industry. In addition, virtually all sensory attributes correlated with instrumental texture parameters, which represents an interesting non-subjective alternative to the sensory analysis for monitoring ripeness. These findings achieved the purposes outlined, which were to verify the behavior of sensory and instrumental textural properties during ripening process, and to evaluate the possible applicability as ripeness predictors. This fact is of particular importance due to the inhomogeneous physiological characteristics of grape seeds at any given date, where the different attribute scores need to be managed to assist in decision-making on the timing of harvest. As a result, the possible implementation of DigiEye^®^ as a routine instrumental tool could be of interest in the viticulture and postharvest sectors to monitor grape seeds maturity, which presents a great challenge to wine research, and may contribute to the improvement of the quality of red wines produced in warm climate regions.

## Figures and Tables

**Table 1:** Parameters extracted from force–distance curve of the texturometer.

Parameter	Abbreviation	Definition
Number of peaks	Np	Number of peaks registered during the assay. For avoiding slight fluctuations in the signal, a smooth on the raw data for eliminating the smallest peaks was applied.
Breaking force (N)	Bf	Force applied on the seed just before breaking. It is a measure of the hardness of the seed.
Breaking decline (N)	Bdc	Force difference the probe exerts just before and after grape seed breaks. It gives an estimation of how brittle the grape seed is.
Elasticity (N/mm)	E	Elasticity of the seed before breaking. It is measured as the slope of the graph from inception until it breaks. To avoid some fluctuations in the beginning of the test, the calculation starts from the point where the force reaches 10 N.
Breaking energy (mJ)	Be	Energy that must be applied to break the seed. It is defined as the area under the curve from the beginning of the test until the break.
Deformation energy (mJ)	De	Energy that must be applied to compress the seed up to 50% of its thickness. It is the area under the entire test curve.
Thickness (mm)	Th	As the test ends at half the thickness, the thickness is calculated by multiplying the distance the probe travels by two.
Breaking distance (mm)	Bd	How much the seed must be deformed to be broken.
Strain index (%)	Si	Relative measure of the breaking distance. Calculated by dividing the breaking distance by the thickness of the seed.
Area ratio (%)	Ar	Ratio between the breaking energy and the deformation energy at 50%.

**Table 2:** General physical and chemical parameters of the Syrah grape sampling ^1^.

Sampling	Weight (g)	Caliber (cm)	Veraison (%)	°Baumé	pH	Acidity (g/L)
25 days BH	1.50 ± 0.13 a	1.15 ± 0.12 a	33 ± 4 a	9.5 ± 0.2 a	2.79 ± 0.07 a	15.23 ± 0.09 a
15 days BH	1.86 ± 0.16 d	1.14 ± 0.15 a	92 ± 9 b	10.3 ± 0.1 b	3.04 ± 0.02 b	9.31 ± 0.21 b
7 days BH	1.72 ± 0.14 c	1.58 ± 0.15 d	92 ± 4 b	11.7 ± 0.3 c	3.50 ± 0.03 c	8.24 ± 0.06 c
H	1.62 ± 0.16 b	1.35 ± 0.11 c	100 ± 4 c	12.3 ± 0.0 d	3.37 ± 0.01 d	7.44 ± 0.04 d
15 days AH	1.53 ± 0.20 a	1.26 ± 0.09 b	100 ± 3 c	14.5 ± 0.1 e	3.74 ± 0.01 e	6.10 ± 0.02 e

^1^ BH, before harvesting; H, harvesting; AH, after harvesting. Different letters in the same column denote significant (*p* < 0.05) differences according to Student–Newman–Keuls test.

**Table 3:** Descriptors used for sensory evaluation of grape seeds ^1^.

Attributes	Definitions	Standard	Sessions	Concentration	Dilutions for Ranking
Vegetal aroma	Green vegetable and stick sensation	*cis*-3-hexenal:IBMP	1st, 2nd	70:50 mg/L	
			3rd	35:25 mg/L	1/2, 1/2.5, 1/3.5, 1/5, 1/10
Bitterness	Characteristic taste of bile, quinine y other alkaloids	Caffeine	1st, 2nd	1 g/L	
			3rd	2.5 g/L	1/1, 1/1.25, 1/1.5, 1/2.5, 1/5
Astringency	Mechanical difficulty in passing the upper lip through the upper incisors after having passed the seed homogenate between the upper lip and incisors	Tannic acid	1st, 2nd	1 g/L	
			3rd	2 g/L	1/1, 1/1.25, 1/1.5, 1/2.5, 1/5
Color	Color evaluation from greenish yellow to brown-green to brown to dark brown to brown-black	Farnsworth–Munsell 100 Hue Test			
Hardness	Force necessary to break the seeds between the molars	High: ripe grape			
Cracking	Auditory sensation that accompanies the total breakage of the seeds	Low: soft, seeds do not crack or break. Medium: seeds break but do not crack. High: seeds break and crack.			

^1^ All solutions were prepared in water.

**Table 4:** Three-way ANOVA results for the evaluation of sensory panel performance.

Interaction	Color	Hardness	Cracking	Vegetal	Bitterness	Astringency
Sample effect	0.000 *	0.000 *	0.000 *	0.000 *	0.000 *	0.000 *
Session effect	0.539	0.982	0.526	0.996	0.726	0.798
Judge effect	<0.001 *	<0.001 *	0.000 *	0.000 *	0.000 *	0.000 *
Sample × session	0.000 *	0.000 *	0.028 *	0.000 *	0.000 *	<0.001 *
Judge × sample	0.348	0.002 *	0.001 *	0.003 *	<0.001 *	<0.001 *
Judge × session	0.997	0.942	0.051	0.957	0.916	0.999

* *p* < 0.05.

**Table 5:** Means and standard deviations of the instrumental textural parameters by maturity stage (MS) established by DigiEye^® 1^.

Textural Parameter	MS1	MS2	MS3	MS4	MS5
Np	3.66 ± 1.39 a	3.92 ± 1.26 ab	4.40 ± 1.48 ab	4.58 ± 1.65 b	4.18 ± 1.29 ab
Bf	50.42 ± 11.30 a	54.24 ± 9.14 ab	58.18 ± 9.67 b	59.82 ± 12.49 b	56.78 ± 14.29 b
Bdc	20.04 ± 8.10 a	20.88 ± 7.18 a	20.21 ± 6.65 a	22.93 ± 9.23 a	18.82 ± 8.66 a
E	98.11 ± 16.90 a	104.92 ± 15.04 ab	105.68 ± 13.20 ab	111.32 ± 18.59 b	103.70 ± 17.84 ab
Be	13.75 ± 5.30 a	14.74 ± 4.16 ab	16.71 ± 5.17 b	16.66 ± 5.20 ab	16.60 ± 6.56 ab
De	36.01 ± 9.08 a	72.93 ± 11.76 b	77.82 ± 14.14 bc	80.74 ± 15.26 c	78.30 ± 17.29 bc
Th	2.19 ± 0.26 a	3.17 ± 0.27 b	3.22 ± 0.26 b	3.24 ± 0.34 b	3.27 ± 0.33 b
Bd	0.53 ± 0.11 a	0.54 ± 0.08 a	0.57 ± 0.09 a	0.55 ± 0.08 a	0.57 ± 0.12 a
Si	0.24 ± 0.05 a	0.17 ± 0.03 b	0.18 ± 0.03 b	0.17 ± 0.03 b	0.18 ± 0.04 b
Ar	0.39 ± 0.13 a	0.20 ± 0.05 b	0.22 ± 0.06 b	0.21 ± 0.07 b	0.21 ± 0.08 b

^1^ Different letters in the same row denote significant (*p* < 0.05) differences according to Student–Newman–Keuls test. Np, number of peaks; Bf, breaking force; Bdc, breaking decline; E, elasticity; Be, breaking energy; De, deformation energy; Th, thickness; Bd, breaking distance; Si, strain index; Ar, area ratio.

**Table 6:** Correlation matrix between instrumental texture parameters and sensory attributes at different maturity stages (MS) established by DigiEye^®^.

	Np	Bf	Bdc	E	Be	De	Th	Bd	Si	Ar	Color	Hardness	Cracking	Vegetal	Bitterness	Astringency
Np	1.00															
Bf	0.99	1.00														
Bdc	0.47	0.42	1.00													
E	0.90	0.91	0.72	1.00												
Be	0.93	0.95	0.13	0.74	1.00											
De	0.83	0.90	0.24	0.83	0.87	1.00										
Th	0.77	0.86	0.18	0.79	0.83	1.00	1.00									
Bd	0.63	0.68	−0.38	0.33	0.87	0.70	0.70	1.00								
Si	−0.73	−0.82	−0.28	−0.82	−0.75	−0.98	−0.99	−0.57	1.00							
Ar	−0.71	−0.80	−0.21	−0.78	−0.76	−0.98	−0.99	−0.61	1.00	1.00						
Color	0.82	0.85	−0.06	0.58	0.96	0.78	0.74	0.91	−0.64	−0.66	1.00					
Hardness	0.86	0.89	−0.03	0.62	0.99	0.80	0.77	0.92	−0.66	−0.68	0.99	1.00				
Cracking	0.84	0.87	−0.06	0.60	0.98	0.81	0.78	0.93	−0.67	−0.69	1.00	1.00	1.00			
Vegetal	−0.78	−0.82	0.14	−0.55	−0.95	−0.80	−0.78	−0.94	0.68	0.70	−0.99	−0.98	−0.99	1.00		
Bitterness	−0.80	−0.84	0.12	−0.57	−0.96	−0.82	−0.79	−0.95	0.69	0.71	−0.99	−0.99	−1.00	1.00	1.00	
Astringency	−0.77	−0.81	0.18	−0.52	−0.95	−0.80	−0.79	−0.97	0.68	0.71	−0.98	−0.98	−0.99	0.99	1.00	1.00

Np, number of peaks; Bf, breaking force; Bdc, breaking decline; E, elasticity; Be, breaking energy; De, deformation energy; Th, thickness; Bd, breaking distance; Si, strain index; Ar, area ratio.

## Data Availability

Data is contained within the article.

## Supplementary Materials

The following are available online at https://www.mdpi.com/article/10.3390/foods10051098/s1, Table S1: Means and standard deviations of colorimetric variables categorized by Maturation Stage.
